# Autonomous Aerial Refueling Ground Test Demonstration—A Sensor-in-the-Loop, Non-Tracking Method

**DOI:** 10.3390/s150510948

**Published:** 2015-05-11

**Authors:** Chao-I Chen, Robert Koseluk, Chase Buchanan, Andrew Duerner, Brian Jeppesen, Hunter Laux

**Affiliations:** Advanced Scientific Concepts Inc., 135 East Ortega Street, Santa Barbara, CA 93101, USA; E-Mails: bkoseluk@asc3d.com (R.K.); cbuchanan@asc3d.com (C.B.); aduerner@asc3d.com (A.D.); bjeppesen@asc3d.com (B.J.); hlaux@asc3d.com (H.L.)

**Keywords:** 3D Flash LIDAR, autonomous aerial refueling, computer vision, UAV, probe and drogue, markerless

## Abstract

An essential capability for an unmanned aerial vehicle (UAV) to extend its airborne duration without increasing the size of the aircraft is called the autonomous aerial refueling (AAR). This paper proposes a sensor-in-the-loop, non-tracking method for probe-and-drogue style autonomous aerial refueling tasks by combining sensitivity adjustments of a 3D Flash LIDAR camera with computer vision based image-processing techniques. The method overcomes the inherit ambiguity issues when reconstructing 3D information from traditional 2D images by taking advantage of ready to use 3D point cloud data from the camera, followed by well-established computer vision techniques. These techniques include curve fitting algorithms and outlier removal with the random sample consensus (RANSAC) algorithm to reliably estimate the drogue center in 3D space, as well as to establish the relative position between the probe and the drogue. To demonstrate the feasibility of the proposed method on a real system, a ground navigation robot was designed and fabricated. Results presented in the paper show that using images acquired from a 3D Flash LIDAR camera as real time visual feedback, the ground robot is able to track a moving simulated drogue and continuously narrow the gap between the robot and the target autonomously.

## 1. Introduction

In-flight aerial refueling was first proposed by Alexander P. de Seversky in 1917 and put into practice in the United States in the 1920s. The original motivation was to increase the range of combat aircraft. This process of transferring fuel from the tanker aircraft to the receiver aircraft enables the receiver aircraft to stay in the air longer and is able to take off with a greater payload. This procedure was traditionally performed by a veteran pilot due to the required maneuvering skills and fast reaction times. In recent years, more and more unmanned air vehicles (UAVs) are used in both military and civilian operations, which motivate researchers to develop solutions to achieve the goal of autonomous aerial refueling (AAR) [1,2,3]. The ability to autonomously transfer and receive fuel in flight will increase the range and flexibility of future unmanned aircraft platforms, ultimately extending carrier power projection [4].

There are two commonly used methods for refueling aircraft in flight: the probe and drogue (PDR) method [5], and the boom and receptacle (BRR) method [6]. The former PDR method is the focus of this paper and is the standard aerial refueling procedure for the US Navy, North Atlantic Treaty Organization (NATO) nations, Russia and China. The tanker aircraft releases a long flexible hose in the PDR method; at the end of the hose is attached a cone-shaped drogue. A receiver aircraft extends a rigid arm called a probe on one side of the aircraft. Because the tanker simply flies straight and allows the drogue to trail behind without making efforts to control the drogue, the pilot of the receiver aircraft is responsible to make sure the probe mounted on the receiver aircraft links up with the drogue from the tanker. This phase is called the approach phase. After the connection is made, the two aircraft fly in formation during which time fuel pumps from the tanker aircraft to the receiver aircraft. This phase is called the station keeping phase, because maintaining a stationary relative position between the tanker aircraft and the receiver aircraft is critical. The final separation phase is completed after the probe is pulled out of the drogue when the receiver aircraft decelerates hard enough to disconnect. One advantage of using the PDR method is that this refueling method allows multiple aircraft to be refueled simultaneously.

The boom and receptacle (BRR) method, on the other hand, utilizes a long rigid, hollow shaft boom extended from the rear of the tanker aircraft. The boom is controlled by an operator who uses flaps on the boom to supervise and direct the boom to the coupling receiver aircraft’s receptacle. The workload of completing the refueling task is shared between the receiver pilot and the boom controller. This method is adapted by the US Air Force (USAF) as well as the Netherlands, Israel, Turkey, and Iran. Although boom and receptacle method provides higher fuel transfer rate and reduces the receiver pilot’s workload, the modern probe and drogue systems are simpler and more compact by comparison. More detailed comparison between these two different operation methods can be found in [2].

There are two required steps in the approach phase before the connection between the receiver and tanker aircrafts can be made—The flight formatting step and the final docking step. The flight formatting step utilizes global positioning systems (GPS) and inertial navigation systems (INS) on each aircraft, combined with a wireless communication system to share measurement information. Modern Differential GPS (DGPS) systems are commonly applied to solve autonomous aerial refueling and they provide satisfactory results in guiding an aircraft to a proximate position and maintaining the close formation between the tanker and receiver [7,8,9,10,11,12]. This technique is not, however, suitable for the final docking step where a physical contact between the probe and the drogue is required. The major challenge is that some aerodynamic effects occur on the drogue and the hose as well as the receiver aircraft itself during the final docking phase. Some of these in flight effects are observed and reported [13,14]. Unfortunately, this dynamic information cannot be captured using GPS and INS sensors because neither sensor can be easily installed on a drogue, which makes the final docking step challenging. Furthermore, the update rate of the GPS system is generally considered too slow for object tracking and terminal guidance technologies that are needed in the final docking step.

Machine vision techniques are generally considered to be more suitable for the final docking task. Many vision based navigation algorithms have been developed for UAV systems [15,16,17,18,19,20]. For aerial refueling, specific developments include feature detection and matching [21,22,23,24], contour method [25], and modeling and simulation [26,27,28,29,30]. In addition to passive imaging methods, landmark-based approaches have also been investigated by researchers. Junkins *et al.*, developed a system called VisNav [31], which employs an optical sensor combined with structured active light sources (beacons) to provide images with particular patterns to compute the position and orientation of the drogue. This hardware-in-the-loop system has been used in several studies [32,33,34] and the results suggest that it is possible to provide high accuracy and very precise six degree-of-freedom position information for real-time navigation. Pollini *et al.* [28,35] also suggest this landmark-based approach and proposed placing light emitting diodes (LEDs) on the drogue and using a CCD camera with infrared (IR) filter to identify the LEDs. The captured images are then served as input information for Lu, Hager and Mjolsness (LHM) algorithm [36] to determine the relative position of the drogue. One major disadvantage of using a beacon type system in the probe and drogue refueling is that non-trivial hardware modifications on the tanker aircraft are required in order to supply electricity and support communication between the drogue and the receiver aircraft.

Martinez *et al.* [37] proposes the use of direct methods [38] and hierarchical image registration techniques [39] to solve the drogue-tracking problem for aerial refueling. The proposed method does not require the installation of any special hardware and it overcomes some drawbacks caused by partial occlusions of the features in most existing vision-based approaches. The test was carried out in a robotic laboratory facility with a unique test environment [40]. The average accuracy of the position estimation was found to be 2 cm for the light turbulence conditions and 10 cm for the moderate turbulence conditions. However, it is well known that traditional vision based technologies are susceptible to strong sunlight or low visibility conditions, such as on a dark night or in a foggy environment. As the 2D image quality declines, the accuracy of the inferred 3D information will unavoidably deteriorate.

It is still a difficult problem to reconstruct 3D information reliably from 2D images due to the inherent ambiguity caused by projective geometry [41,42,43] and the benefits of using 2.5D information in robotic systems for various tasks have been documented [44,45]. For probe-and-drogue autonomous aerial refueling application specifically, using a time-of-flight (ToF) based 3D Flash LIDAR system [46,47] to acquire information of the drogue in 3D space has been proposed in Chen and Stettner’s work [48]. They utilized the characteristics of the 2.5D data sensor provided and adopted a level set method (LSM) [49,50] to segment out the drogue for target tracking purposes. Because of the additional range information associated with each 2D pixel, the segmentation results become more reliable and consistent. The indoor experiments were carried out in a crowed laboratory, but the detected target showed promising results.

There are two major challenges for the final docking (or hitting the basket) step in the probe-and-drogue style autonomous aerial refueling: (1) the ability to reliably measure the orientation as well as relative position between the drogue trailed from the tanker aircraft and the probe mounted on the receiver aircraft and (2) advanced control systems to rapidly correct the approaching course of the receiver aircraft to ensure the eventual connection between the probe and the drogue. This paper offers a potential solution to the former difficult task. Although some design descriptions of the ground test robot are also presented, the intent is only to evaluate the proposed method in a more practical experimentation. We encourage readers who are interested in navigation and control aspects of the unmanned systems to consult more domain specific references, such as [51,52,53]. This paper employs a 3D Flash LIDAR camera as the source of the input data, but differs from [48] in that this paper suggests a sensor-in-the-loop method incorporating both hardware and software elements. In addition, a ground feasibility test was performed to demonstrate the potential for in air autonomous aerial refueling tasks.

## 2. Method

The method section is organized as follows. Descriptions of the sensor employed for data acquisition is first introduced in Section 2.1. Reasons for choosing this type of sensor over other sensors are discussed in depth. Section 2.2 presents characteristic analysis results of a real drogue for aerial refueling task. Section 2.3 briefly described how the 3D Flash LIDAR camera internally computes range information followed by the discussions of a more forgiving drogue center estimation method in Section 2.4.

### 2.1. 3D Flash LIDAR Camera

**Figure 1:**
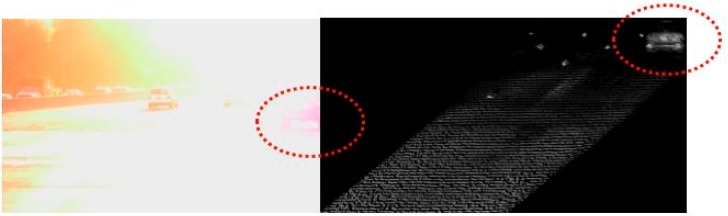
2D camera *vs.* 3D Flash LIDAR camera.

A 3D Flash LIDAR camera is an eye safe, time-of-flight (ToF) based vision system using a pulsed laser. Because the camera provides its own light source, it is not susceptible to lighting changes, which are typical challenges for traditional vision based systems. Figure 1 illustrates the comparison between a regular 2D image on the left and an image acquired from the 3D Flash LIDAR camera on the right under a strong sun light condition.

This camera is, however, similar to a traditional 2D camera that uses a focal plane array with 128 × 128 image resolution. The only difference is that a 3D Flashed LIDAR camera provides additional depth information for every pixel. Each pixel triggers independently and the associated counter for the pixel will record the time-of-flight value of the laser pulse to the objects within the field of view (FOV) of the camera. Because of this similarity, the relationship between a 2D point and the 3D world can be described using a commonly used pinhole model as shown in Equations (1) and (2) below [43].

(1) $$x=KR[\;I\;|-\tilde{C}\;]X$$

(2) $$K=\left[\begin{array}{ccc} f & 0 & p_{x}\\ 0 & f & p_{y}\\ 0 & 0 & 1 \end{array}\right]$$

The upper-case *X* is a 4-elements vector in a 3D world coordinate frame while the lower-case *x* is a 3-elements vector in the 2D image coordinate system. *K* is the internal camera parameter matrix with focal length *f* and principle point (*p_x_*, *p_y_*) information. *R* represents a 3 × 3 rotation matrix together with $\tilde{C}$ are called the external parameters which relate the camera orientation and position to the world coordinate system. Converting each pixel into 3D space is a straightforward task that requires simple geometric equations when the 3D Flash LIDAR camera is used because the depth information is available and therefore complicated mathematical inference is no longer needed. Moreover, it is possible to construct geo-reference information for every point if the global coordinates of the camera are known because the calculated 3D positions are relative to the camera center.

**Figure 2:**
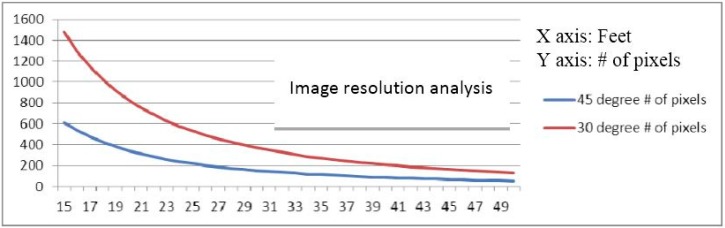
Range *vs.* resolution analysis, Number of pixels that will be illuminated on a 27-inch (68.58 cm) diameter object from 15 feet (4.57 m) to 50 feet (15.24 m).

Another common property both types of cameras share is that they can easily change the FOV by choosing different lenses. For a fixed resolution image, it is expected to observe more details when a narrower FOV lens is selected as shown in Figure 2. The total number of pixels that will be illuminated on a known object at a certain distance can be estimated. The blue curve in Figure 2 represents the case of a 45° FOV lens, while the similar curve in red represents a 30° FOV lens. All of these 2D pixels detected by the camera can be uniquely projected back in 3D space for position estimates and the process does not require additional high quality landmarks. Figure 2 also shows rapid growth of the total number of pixels in the images as the distance between the target and the camera decreases.

**Figure 3:**
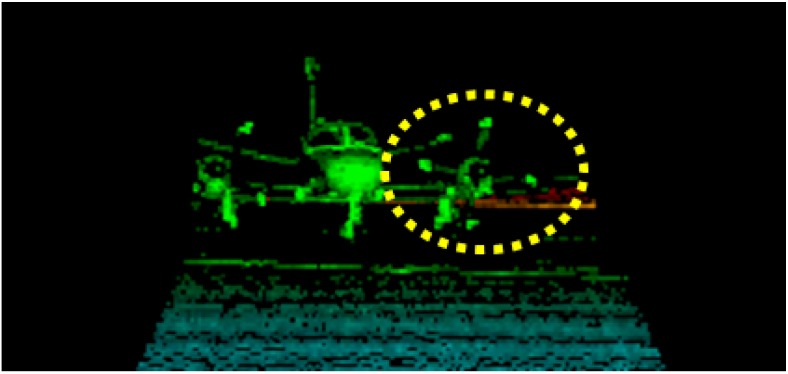
Propeller tips move at 220 meters per second without motion blur.

**Figure 4:**
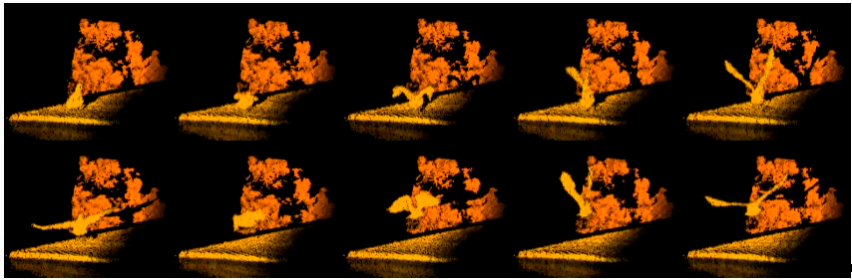
A seagull is taking off from a rooftop.

Unlike a traditional scanning based lidar system, a 3D Flash camera does not have moving parts. All range values for the entire array are computed after only one shot of laser pulse. This camera is therefore capable of capturing images of a high-speed moving object without motion blur, which is particularly important for the autonomous aerial refueling application. Figure 3 shows the propeller of an airplane rotating at 220 meters per second, which is frozen by speed-of-light imaging. Figure 4 shows a seagull taking off from a roof in consecutive frames of motion. As can be seen, each snap shot is a clean image. Furthermore, as the 3D Flash LIDAR sensor shares so many common properties with conventional CCD cameras, many existing computer vision based algorithms, libraries and tools can be adapted to help solving traditionally difficult problems with comparatively minor modifications. OpenCV (Open Source Computer Vision) [54], for example, is one of the most popular libraries in the computer vision field of research and the PCL (point cloud library) [55] for both 2D and 3D point cloud data processing. As for autonomous systems, Robot Operating System (ROS) is a collection of software frameworks [56] and a useful resource for researchers since machine vision is an essential component for robots as well.

### 2.2. MA-3 Drogue

**Figure 5:**
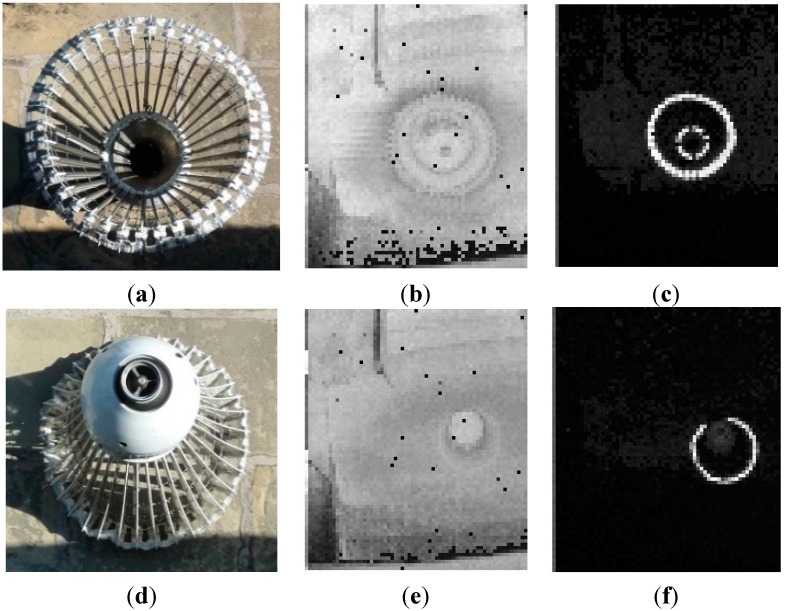
Strong retro-reflective signals from the drogue. (**a**–**c**) simulate the images perceived by the receiver aircraft; (**d**–**f**) simulate the images perceived by the tanker.

Instead of passively using the default settings in the 3D Flash LIDAR camera, experiments were carried out to explore proper settings for the drogue detections task and invaluable information was acquired using a real Navy drogue from PMA 268. The experimental results show the drogue contains retro-reflective materials and it was fortunate in terms of detecting the drogue at all needed distances. Figure 5 summarizes the experimental results. The drogue was facing up and located on the ground. A 3D Flash LIDAR camera was set up about 20 feet (6.1 m) above the drogue on our 2nd floor balcony, facing down perpendicularly. Figure 5a–c mimic images that would be observed from the receiver aircraft. Figure 5a is a regular 2D color image for visual reference purpose and Figure 5b is the intensity image captured by the 3D Flash camera. Figure 5b,c are the same images except the laser energy in Figure 5c is only 0.01% of that in Figure 5b after a neutral density filter is applied. The same strong retro reflective signals are also observed when switching the view point from the receiver aircraft to the tanker side as shown in Figure 5d–f. Although the majority of research related to the probe-and-drogue style autonomous refueling focuses on simulating scenarios of mounting sensors in the receiver aircraft. The possibility of equipping sensors on the tanker side has also been considered. This experiment is designed to help us understand what can be expected from the sensor output under different parameter settings and raise a flag if some limitations are found. Fortunately, there are no obvious show stoppers for either option in terms of received signals.

To limit the scope of this paper’s discussion, the assumption of observing the drogue from the receiver aircraft is made. It is crucial to balance the laser power and the camera sensitivity setting to achieve the most desired signal return level. One of the challenges in the system design lies in satisfying two extreme cases in the autonomous aerial refueling application: (1) the laser must generate enough power to provide sufficient returns from the drogue when it is at the maximum required range; and (2) when the drogue is very close to the camera as expected in the final docking step, a mechanism to avoid saturating the acquired data (due to the powerful laser), is also mandatory. Based on the observation described earlier, the first extreme case does not seem to be a concern at anymore. All efforts should be dedicated to solve the second extreme scenario.

### 2.3. Range Calculation

How a 3D Flash camera provides ready to use range information is briefly discussed in this section. Target range measurement, based on time-of-flight of the laser pulse, is determined independently in each unit cell. With a high reflectivity target, such as retro-reflective materials in the autonomous aerial refueling application, the return amplitude can be saturated. In this saturated case, the time-of-flight can be interpolated. The saturation algorithm is certainly suitable for the close up scenario when the probe of the receiver aircraft is about to make a connection with the drogue. However, the detected signals from the drogue may not always be highly saturated when the refueling process starts from some distance away, since the laser energy follows an inverse-square law. Even a retro-reflective drogue may look like a low reflectivity target when the distance between the target and the camera is large. To include this non-saturating case, the time-of-flight can also be interpolated from the non-saturated signal. To achieve the best of both worlds, a 3D Flash LIDAR camera optimizes the non-saturating and saturating algorithms. The optimized algorithm is implemented in the camera’s field-programmable gate array (FPGA) for real time output.

### 2.4. Drogue Center Estimation

The ability to reliably measure the relative position and orientation between the drogue trailed from the rear of the tanker aircraft and the probe equipped on the receiver aircraft is one of the main challenges in the autonomous aerial refueling application. In the previous work [48], a level-set front propagation routine is proposed for target detection and identification tasks. Together with sufficient domain knowledge to quickly eliminate unlikely target candidates, the proposed method provides satisfactory results in estimating the center of the drogue after all 3D points on the drogue are identified. Any computation related to the relative position and orientation becomes straightforward when the center point in 3D space is established.

**Table 1:** Domain knowledge table.

Item	How We Can Use This Information
Single object tracking	Cross over issue is not considered
Single camera	Information handling is simplified
Simple background	Only have probe, drogue, hose, and tanker
Plane movement	Drogue randomly moves in horizontal/vertical directions
Known object of interest	Highly reflective materials. Camera setting can be simplified
Bounded field of view	Use automatic target detection and recognition for each frame instead of tracking which will fail if the target is outside the FOV.

**Figure 6:**
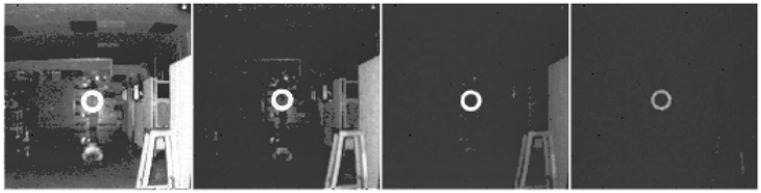
Adjusting camera setting.

After learning more about the characteristics of the real drogue discussed in Section 2.2, the domain knowledge references are updated and summarized in Table 1. One important piece of information, which was missing in the previous work [48], is that a drogue appears to contain high reflective materials at least to the wavelength a 3D Flash LIDAR camera detects. The first rational idea for a sensor-in-the-loop approach would be taking advantage of this fact. By lowering the camera gain, a 3D Flash LIDAR camera will detect only strong signal returns from highly reflective materials such as a refueling drogue. Figure 6 shows a few snap shots from a video sequence while lowering the camera gain continuously. As can be seen, by gradually applying these changes (from left to right), a crowded lab disappears in the final frame and only the high reflectivity target remains. This simple adjustment in the camera makes the subsequent analysis much easier because there are fewer pixels left to process and the majority of these remaining pixels are on the target of interest. Therefore, less computational cost can be expected while the confidence level of the detected target increases because there is not much room for an image processing algorithm to make a mistake. This is the essence of adapting a sensor-in-the-loop approach when solving a difficult problem. Data acquisition and image processing are often coupled together but treated as two separate components in a system pipeline. While it is convenient to isolate individual components for discussion purposes, global optimization from the total system point of view most likely cannot be achieved without considering both components simultaneously.

Is object tracking is really necessary; or is the automatic target detection and recognition (ATD/R) all we need? The ATD/R module is essential for most of the automatic tracking systems and is usually engaged in either the initialization stage or the recovery stage where establishing the target of interest is required. Given the fact that a 3D Flash LIDAR camera, like a traditional 2D camera, can only image objects within its FOV, when the target drifts out of the FOV and then reappears later, an ATD/R component is required to reinitialize the target. A tracking process implicitly assumes that the target appearing in the current frame would be located somewhere close to where it was in the previous frame. Therefore, the tracking algorithm is designed to narrow the searching space to limit the computational cost. In the previous work [48], a level set front propagation algorithm is used to track the target. Existing information such as the silhouette of the target and the estimated center in the previous frame is used for seed point selection to efficiently identify the target. A tracking process does not seem to be required when the target of interest can be reliably identified with proper camera settings as shown in Figure 6.

As Figure 5c clearly illustrated, a drogue has retro-reflective materials on both the canopy (the outer ring) and the center rigid body structure (the inner ring). Although some distortion might be expected from the canopy of the drogue, it usually forms a circle thanks to aerodynamic flow. Many research papers have been published on circle fitting [57,58,59,60,61,62,63,64,65]. In general, the basic problem is to find a circle that best represents a collection of *n* ≥ 3 points in 2D space (image coordinate system) labeled (*x*_1_, *y*_1_) (*x*_2_, *y*_2_), …, (*x_n_*, *y_n_*) with the circle equation described by (*x* − *a*)^2^ + (*y* − *b*)^2^ = *r*^2^ and we need to determine the center (*a*, *b*) and radius *r*. One reasonable error measure of the fit will be given by summing the squares of the distance from the points to the circle as shown in Equation (3) below.

(3) $$SS\left(a,b,r\right)=\overset{n}{\underset{i=1}{\sum }}{\left(r-\sqrt{{\left(x_{i}-a\right)}^{2}+{\left(y_{i}-b\right)}^{2}}\right)}^{2}$$

Coope [59] discusses numerical algorithms for minimizing SS (sum of squares) over *a*, *b*, and *r*. With various ways of formulating the same problem, each circle fitting algorithm results in different accuracy, convergence rate and tolerance level for noise. The goal of this paper is not to develop a new algorithm to determine the center of the circle, but to evaluate and select one algorithm that can reliably and efficiently output the center of the drogue when an image frame from 3D Flash LIDAR is presented. Utilizing these well-studied algorithms, we expect to enlarge the effective working area beyond the FOV boundary because these algorithms can estimate the center of a circle even if the circle is partially occluded.

**Figure 7:**
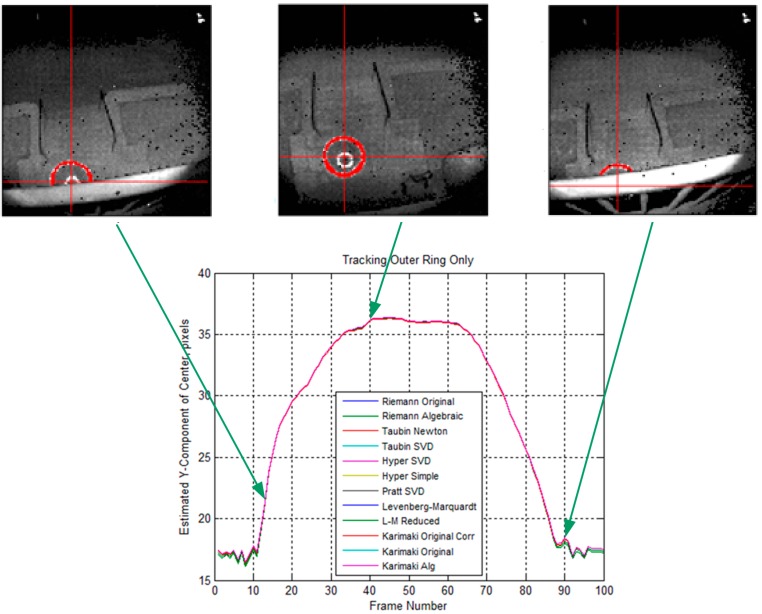
Comparison study of different circle fitting algorithms by using only the outer ring.

Figure 7 summarized the evaluation results using the real target—A MA-3 drogue. The drogue was oriented vertically upward on the ground 20 feet (6.1 m) below the second floor balcony as shown in the middle intensity image of Figure 7. The 3D Flash LIDAR camera was moving in toward the balcony while facing down perpendicularly to create images of partially occluded drogue for testing as shown in the left and the right intensity images of Figure 7. These images of the drogue have been manually segmented and processed using 12 circle fit algorithms with Chernov’s Matlab implementation [58,66]. Over the 100 frames of the sequence, the initial images of the drogue are occluded by the railing of the balcony, producing a partial arc. As the camera moves, the arc becomes a complete circle. The sequence ends with the camera returning to the initial origin.

The upper plots from left to right are the intensity images acquired from the 3D Flash LIDAR for frames 13, 40 and 90. Pixels segmented for the outer ring only are shown in red, and the red cross-hair shows the estimated center using the Taubin SVD algorithm [65]. Since the primary movement is in the y-direction, the lower plot shows the estimated Y component of the ring’s center *vs.* frame number for each of the 12 fit algorithms indicated in the legend. The plot shows very close agreement of all 12 algorithms with the real drogue data, including those frames where part of the ring is occluded by the railing on the balcony. While the ground truth of the actual center is not available, the estimated center in the intensity image appears subjectively to be reasonably accurate. Barrel distortion of the receiver lens is evident in the railing, but not particularly noticeable in the ring image or the estimate of the ring’s center.

**Figure 8:**
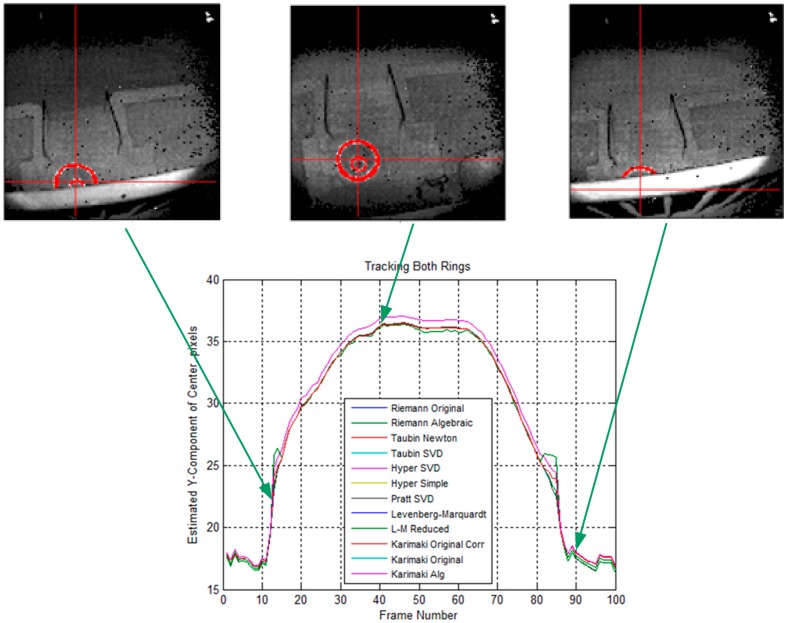
Comparison study of different circle fitting algorithms by using both inner and outer rings.

However, the close agreement conclusion does not hold when both inner and outer rings are used. Figure 8 shows the comparison study results using the same sequence. Again, the segmentation is performed manually. The plot shows that some of the algorithms were affected more than others having both rings present and, similarly, some algorithms were more adept at handling the appearance of the inner ring. The algorithms that were upset by the appearance of the inner ring were the Levenberg-Marquardt [67,68,69] and Levenberg-Marquardt Reduced algorithms (both are iterative geometric methods) and the final two Karimaki algorithms [61], lacking the correction technique. All of the other algorithms agreed closely and handled the appearance of the inner ring very well. Based on these analysis results, a Newton-based Taubin algorithm is selected and implemented in the ground navigation robot.

Three major changes improve the overall robustness of the drogue center estimation process. (1) Pixel connectivity is no longer required. The level set front propagation algorithm proposed in the previous work [48] implicitly assumes the target of interest is one connected component. If this assumption fails in practice scenarios, the analysis for subsequent segmentation and for detecting and extracting the target automatically will be unavoidably complicated. In contrast, the circle fitting algorithms, by design, perform well on disconnected segments or even on sparse input pixels; (2) Partially out of FOV cases are handled naturally. Additional domain knowledge and heuristics need to be incorporated into the previously proposed method for the segmentation routine to handle partially out of FOV cases reliably. The circle fitting algorithms, on the other hand, handle these cases without any special treatments. As can been seen in Figure 7 and Figure 8, a small segment of an arc is all these algorithms require to predict the circle center; (3) Estimation of the target size in advance is not required. Given the FOV of a camera as well as the distance between a known object and the camera, it is possible to estimate the total number of pixels in each image that will be illuminated on this object as shown in Figure 2. Such information is very important for the previously proposed segmentation routine to quickly eliminate the unlikely candidates. It is, however, not applicable to the circle fitting algorithms because observing the entire target is not required. A reliable estimation requires careful attention to outliers. The common outlier removal algorithm, random sample consensus (RANSAC) [70], can be integrated into two different stages of the proposed method—When the center of the target in 2D image is estimated and when the final output range/depth of the target center in 3D space is estimated.

With all the benefits described above, this paper suggests a more fault tolerant drogue center estimation method, which combines camera sensitivity setting with a circle fitting algorithm. This fault tolerant capability is desirable in a practical system, which is expected to handle challenging scenarios, such as imaging a used drogue that may be covered with spilled fuel, estimating the center of the drogue when it drifts partially out of FOV, and processing images with pixel outages.

## 3. Ground Test Evaluation

We evaluate the proposed sensor-in-the-loop method through a ground test. The goal of this autonomous aerial refueling ground test is three fold: (1) demonstrate the proposed method that combines the sensitivity setting of a 3D Flash LIDAR camera with computer algorithms is able to successfully provide information for terminal guidance; (2) the ground test should be performed in real time, not much extra computational power is required because the camera is doing most of the range calculation; and (3) the ground test should be completed autonomously.

To achieve these three goals, a small ground navigation robot is designed and fabricated due to lack of off-the-shelf options specifically for autonomous aerial refueling evaluation. This section is organized as follows: the design idea as well as the capabilities of the robot is discussed in Section 3.1. As we mentioned earlier, the control electronics, although briefly discussed in Section 3.2, is not the focus of this paper. The mock up drogue is described in Section 3.3, followed by the pseudo codes in the robot. Finally, the experimental results and evaluation as well as discussion are shown in Section 3.5 and Section 3.6, respectively.

### 3.1. Robot Design and Fabrication

For demonstration purposes, this robot possesses three types of simplified motions: X (left-right), Y (forward-back), and Z (up-down). At first glance, allowing the robot to move in the Y-direction is simple, only a drivetrain and a platform are required. Movement in the X-direction can be achieved by having two drive motors that each independently controls wheels on the left and right side. From the point of view of the camera, however, this does not properly simulate the motion of the refueling aircraft. Similar to changing lanes on the freeway in a ground vehicle, the aircraft most likely maintains forward-looking direction when it moves side-to-side or up-and-down with very little rotation. Rotation along Y-axis is not applicable in a ground test. To stay within the scope of the goal, the robot has a pivoting turret, allowing the camera to face forward, while the base steers left and right. This requirement adds little complexity, as a stock ring-style turntable paired with a motor and drive belt allow the camera to pivot as needed.

**Figure 9:**
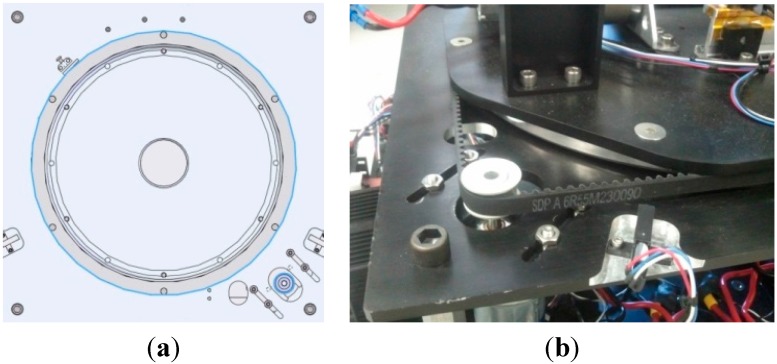
(**a**) Cross section view of the turntable in the virtual model; (**b**) Actual image of the interaction of pulley and belt.

The Z-direction requires a mechanism that is able to raise and lower the camera with both control and stability because the actual height of the camera is important in the ground test. The robot requires the exact amount of traveling distance for each movement. The requirement also calls for a large traveling range, approximately 20 inches (50.8 cm), in the Z-direction. A scissor lift powered by a lead screw design is selected for its capability of control and stability, along with being compact and having a large traveling range. For the goal of facing forward as discussed earlier, a ring-style turntable is attached to the bottom, allowing the scissor lift to pivot freely. Motion control is achieved by adding a timing belt wrapped around the outside of the turntable and held securely with a setscrew; then a pulley attached to a motor is also mated to the belt to give the system motion. The pulley motor assembly serves an additional purpose of applying tension to the belt by being mounted on slots. Figure 9 shows the virtual model’s cross sectional view of the turntable and the actual image of how the pulley and belt interact.

**Figure 10:**
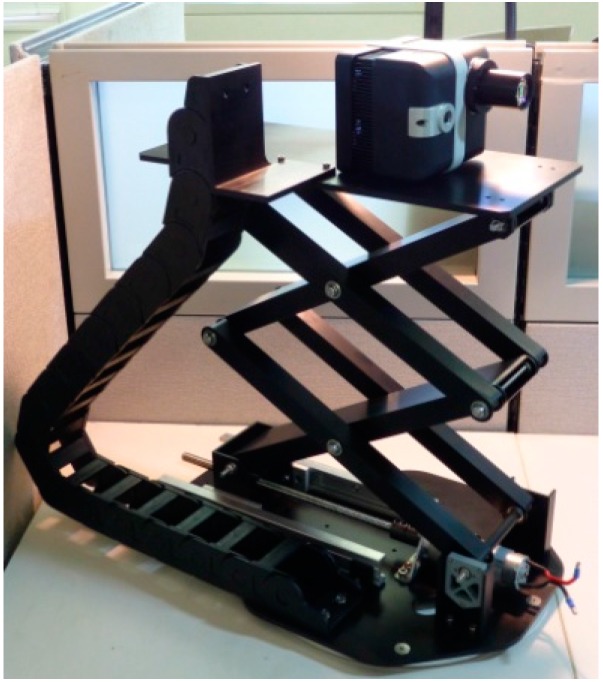
Completed scissor lift assembly with the 3D Flash LIDAR camera at the top.

The scissor lift is a crucial component for this robot, as it took up the majority of design time and fabrication cost. The end result is a functional lift that is capable of rising 20 inches (50.8 cm) in a few seconds at maximum speed and can also sit at a compact minimum position. To group all connecting wires between the 3D Flash LIDAR camera at the top of robot and control electronics at the base, E-chain is designed and fabricated as one of the essential pieces of the scissor lift. In addition to gathering all of the wires and isolating them from moving parts in the robot, the E-chain also avoids excess wire bending with its minimum bend radius, reducing wire fatigue due to the lift moving from high to low positions. Figure 10 shows the completed scissor lift assembly with the 3D Flash LIDAR camera.

### 3.2. Control Electronics

Talon SR speed controllers are used to control motors of the robot when they receive command pulses from an off-the-shelf Arduino Mega micro-controller. Both Z (lifting motion) and Theta (pan motion) axes have limit switches to prevent the motor from traveling beyond the designed 180° boundary. Position feedback for each axis was provided by a rotary encoder. The Arduino micro controller uses the encoder position for speed regulation and position tracking. Unlike many expensive high-end motion controllers that apply proportional-integral-derivative (PID) control or use an S-Curve like pattern generator to provide soft starting and stopping motion by gently increasing or decreasing the speed gradually, the Arduino provides only a triangular waveform. It is, however, sufficient for this ground test demonstration if some fuzzy logic is incorporated in the applied triangular curve pattern to prevent jerky motions.

**Figure 11:**
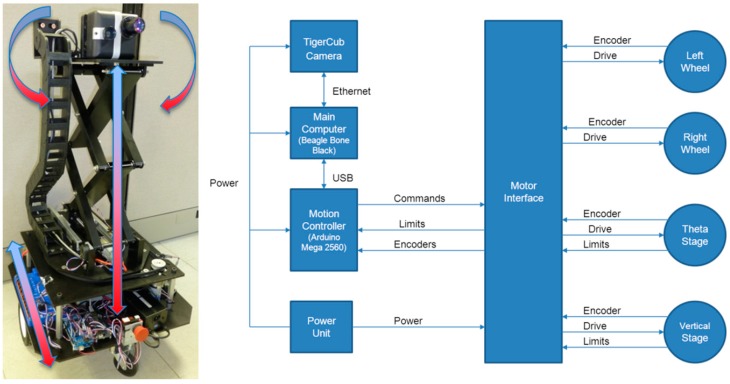
Ground navigation robot (**left**) and its system block diagram (**right**).

Also, to demonstrate that fairly little computational power is required to complete the task, a popular commercial off-the-shelf (COTS) single board processor, Beagle Bone Black, is chosen to handle all higher level decision making processes. The Beagle Bone Black is responsible for receiving range and intensity images from the 3D Flash LIDAR camera via Ethernet and in real time performing drogue center estimation proposed in this paper. Finally, serial commands derived from the relative drogue position need to be sent to the COTS motion controller, Arduino, to achieve the desired motions. Concurrent motions such as moving both wheels in unison can be accomplished by sending commands for both axes and then executing them simultaneously. Figure 11 shows the final ground navigation robot and its system block diagram.

### 3.3. Full Size Drogue Mock-up

**Figure 12:**
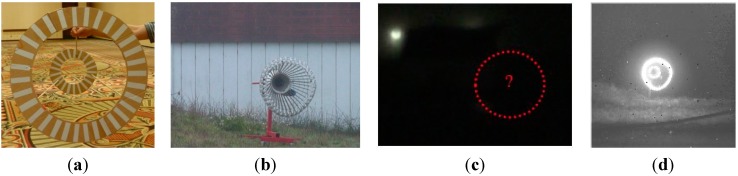
The full size drogue mock up.

For the purpose of maneuvering the drogue target easily during the ground test, a full size drogue mock-up is built from cardboard and retro reflective tape strips as shown in Figure 12a. This simulated drogue consists of two concentric cardboard rings connected by three light weight wooden rods to mimic the outer parachute ring and the center rigid body portion of the real drogue as shown in Figure 12b. Figure 12b shows a real MA-3 drogue mounted on an engine stand and the outer parachute ring was expanded by stiff wires to create a profile similar to that expected during the aerial refueling task. The picture was taken on 14 November 2012 in Eureka, California, which is noted for heavy fog during the winter. One data sequence was captured earlier on that day at 3:23 a.m. The drogue was located in front of the small shed and was about 60 feet (18.29 m) away from the 3D Flash LIDAR camera.

The visible image in Figure 12c appears dark and blurry due to the foggy condition at the time while the intensity information acquired from a 3D Flash LIDAR camera shows two very distinct retro-reflective rings. This encouraging observation suggests that a 3D Flash LIDAR camera, together with the proposed center estimation method, has potential to provide terminal guidance information in the autonomous aerial refueling application, even in degraded visual environments (DVE) such as fog and cloud. This idea requires more rigorous experiments and the discussion is beyond the scope of this paper.

### 3.4. Pseudo Codes Implemented on the Ground Robot

1. Loop Until ***Distance*** < ***Distance_Threshold***
2. Input one 128 × 128 3D Flash LIDAR image ***A***
3. ***Final_list_count*** = 0;      //initialize
4. ***Distance*** = 0;          //initialize
5. ***Final_list*** = { };          //initialize
6. For each pixel ***p*** with quadruplets information—(x, y, range, intensity) in ***A*** {
7. if (the intensity of ***p*** > (range associated) minimum intensity threshold){
8.       Add ***p***(***x***, ***y***, **1**/***range***) into ***Final_list***
9.       ***Final_list_count++;***
10.       ***Distance*** + = ***p***’s range;
11.    }
12. } End For
13. if (***Final_list_count*** < 10) continue;
14. ***Distance*** = ***Distance***/***Final_list_count***;
15. ***Estimated_Center*** = Taubin_Circle_Fit (***Final_list***)
16. Ground_Robot_Motion (***Estimated_Center, Distance***)
17. End loop

The above pseudo code illustrates how the ground navigation robot processes input images acquired from a 3D Flash LIDAR camera and calculates necessary information to carry out its next move. As can be seen, it is not a tracking algorithm, instead this algorithm performs center estimation and distance computation on every individual frame without using any information from previous frames. The robot will stop moving after the computed distance value is smaller than a pre-determined threshold (Line 1). All the thresholds in this pseudo code are adjustable and are expected to be changed in the flight test once the final configuration is determined.

An image from the 3D Flash LIDAR is a 128 by 128 array with co-registered range and intensity information for every pixel. To speed up the execution time, a prescreening is performed from Line 6 to Line 12. Only pixels with high enough intensity values will be kept for subsequent processes. Each selected pixel contributes its (*x*, *y*) coordinate information as well as the weight for Newton-based Taubin algorithm [65] to estimate center in Line 15. Experimental results suggest that using 1/range weighting formula in Line 8 to separate the outer canopy portion of the drogue from the center rigid body part of the drogue is beneficial. The robot is designed to keep the estimated drogue center on the center of the feedback image acquired from Flash LIDAR. Horizontal and vertical deviations in either x- or y-axes will trigger robot movements, such as turn and height adjustment, in Line 16.

### 3.5. Perform Ground Test Autonomously

To perform the ground test at an undisturbed location, a large conference room measuring 90 feet (27.43 m) × 44 feet (13.41 m) is used. The robot and the simulated drogue target are set up in opposite corners of the room facing each other to establish the 100 feet (30.48 m) travel distance configuration. According to the report of Autonomous Airborne Refueling Demonstration (AARD) project [71], we believe the selected venue with 100 feet (30.48 m) length is a representative and sufficient setup to evaluate the sensor. The process consisted of a Trail position, a Pre-Contact position and a Hold position. The Trail position is for the refueling aircraft to initialize the rendezvous for the closure mode and it is located at 50 feet (15.24 m) behind the Pre-Contact position. A Pre-Contact position is at 20 feet (6.1 m) behind the drogue where a closure rate of 1.5 feet (0.46 m)/s is used to capture the drogue. After the drogue is captured, the closure velocity of the receiver aircraft is reduced as the aircraft continues forward to the Hold position. The Hold position is normally 10 feet (3.05 m) ahead of the average drogue location. The normal traveling distance to complete this process is 80 feet (24.38 m).

**Figure 13:**
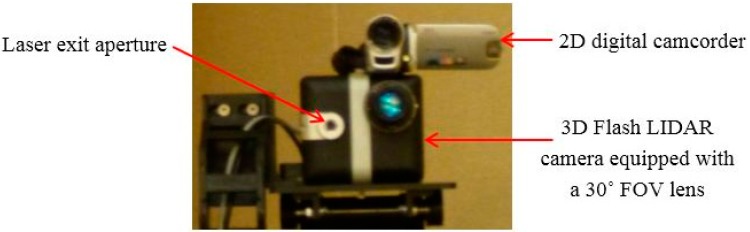
2D digital camcorder and 3D Flash LIDAR camera.

A digital camcorder was placed on top of the 3D Flash LIDAR camera to record visible videos at the same time, as shown in Figure 13. The output images from the Flash LIDAR camera are stored in the secure digital (SD) memory card on the Beagle Bone Black processor. Please note that no careful alignment has been performed in synchronizing frames from the two cameras during this test. The main purpose of the 2D camcorder is to provide feedback for intuitive reference. Spatial alignment between 2D and 3D images is not the focus of this ground test either, because various lenses with different FOVs, 9°, 30°, and 45°, are evaluated.

**Figure 14:**
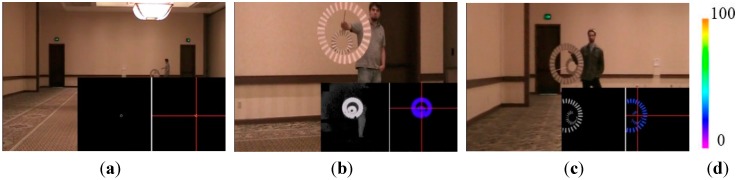
Ground test experimental results using various FOV lenses: (**a**) 45° FOV lens; (**b**) 30° FOV lens; and (**c**) 9° FOV lens; (**d**) Color palette from 0 feet (0 m) to 100 feet (30.48 m).

Figure 14 shows some snapshots of the ground test results. Each image consists of three pictures—A regular visible 2D picture from the camcorder superimposed by two small lidar images at the bottom right corner. The left small lidar image is the original intensity image from the 3D Flash LIDAR camera given a proper sensitivity while the right small image displays pixels actually used in the center estimation process. The red cross-hair highlights the computed center in 2D image space and the range-color-coded drogue visualizes the distance. The color palette for range indication, from 0 feet (0 m) to 100 feet (30.48 m), is also included in the Figure 14 where orange represents the farthest distance of 100 feet (30.48 m).

**Figure 15:**
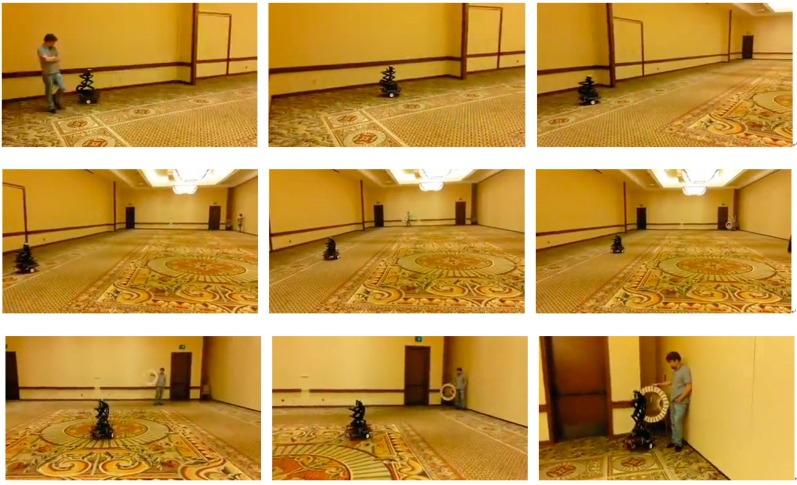
Autonomous aerial refueling ground test demonstration.

As can be seen in Figure 14a, the simulated drogue located at about 90 feet (27.43 m) away appears to be very small in 3D Flash LIDAR imagery when a 45° FOV lens is used. This observation suggests a narrower FOV lens would be beneficial as the analysis result concluded earlier in Figure 2. A range dependent intensity threshold was applied to quickly exclude some non-target pixels, resulting in more accurate drogue center estimation as shown in Figure 14b. The final experimental result in Figure 14c illustrates what can be expected if a 9° FOV lens and only 1% of the laser energy is supplied. As can be seen, only the retro-reflective tape is visible in this configuration. Valuable lessons are learned to better adjust the parameters in this integrated ground test system. At the end of this test, all three objectives have been successfully achieved. Figure 15 shows nine consecutive snapshots of one test run.

### 3.6. Evaluation and Discussion

**Figure 16:**
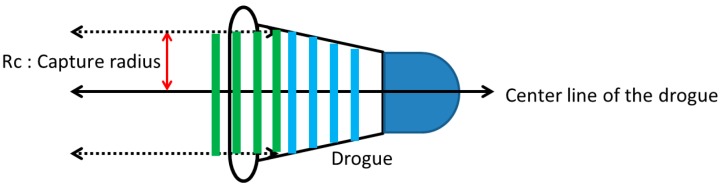
The catch criteria.

In the probe-and-drogue refueling process, it is not uncommon for the drogue to make contact with or possibly cause damage to the receiver aircraft. For system safety analysis purposes, miss and catch criteria were imposed in the Autonomous Airborne Refueling Demonstration (AARD) project [71]. The concept of the catch criteria, as shown in Figure 16, are sensible evaluation options to be adapted in this ground test. The capture radius, Rc, suggested by the project pilot with a 90 percent success rate and was defined as being 4 inches (10.16 cm) inside the outer ring of the drogue. In a successful capture, the probe must remain within the zone with green stripes, a tube coaxial to the drogue defined by Rc, and transition into the zone with blue stripes during the hold stage.

**Figure 17:**
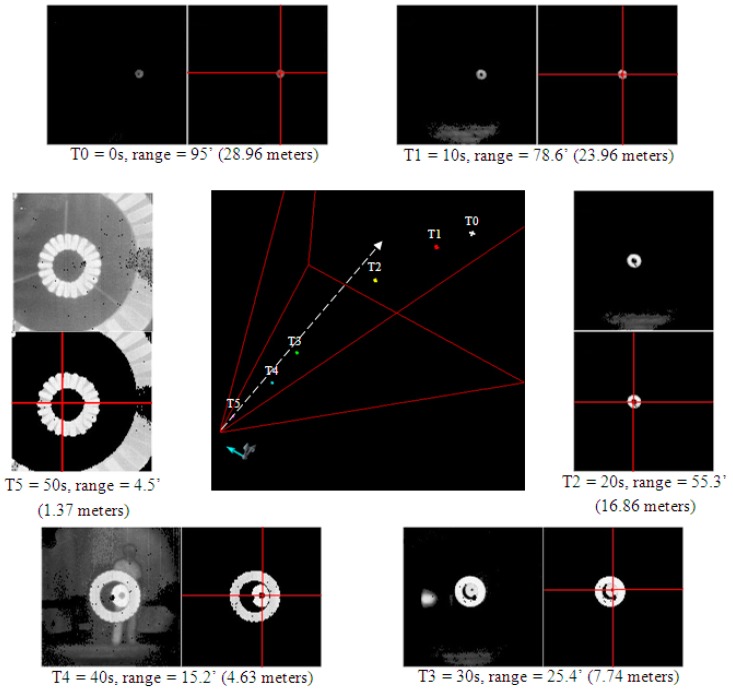
Experimental results from a successful catch run.

In the real operating scenario, the 3D Flash LIDAR camera is most likely to be rigidly mounted close to the probe on the receiver aircraft. As the 3D point clouds generated from the camera data are all relative to the focal point of the camera, the relationship describing the point clouds and the tip of the prober will be standard 3D rotation and translation matrices, like regular rigid body transformations. Without loss of generality, the following data set in Figure 17, from the same sequence that generated snap shots in Figure 15, will demonstrate a successful catch.

Figure 17 consists of seven subfigures. The center subfigure shows a pyramid shape area representing the enclosed space in 3D, which can be observed by the 3D Flash LIDAR camera with a 30° FOV lens. The tip of the pyramid is where the camera is located and the dashed arrow from the tip shows the direction this camera is pointing. Within the pyramid, six points in 3D space are shown with different colors and associated time stamped labels. Detailed information of these six points are displayed in a circle around the center subfigure. In the top left corner, a pair of intensity images captured by the camera in the beginning of the test run at T0 (0 s). The image on the left shows the original input data while the other shows the data actually feed into the center estimation routine after filtering, as described in Section 3.4 Pseudo Codes Line 7. The red cross-hair represents the estimated center with estimated range equaling 95 feet (28.96 m). The range estimation is a simple average computation as shown in Pseudo Codes Line 10 and Line 14, which guaranties a bounded number between the outer canopy and the inner of the rigid body of the drogue. The 3D point is displayed in white labeled T0 in the center subgraph.

In clockwise order, the top right corner shows the image pair acquired at T1 (10 s) with estimated range equaling 78.6 feet (23.96 m). As can be seen in T2 (20 s) data with a 55.3 feet (16.86 m) range, the observed target becomes brighter due to the laser energy at the closer range (inverse-square law). With the sensor-in-the-loop approach, the camera parameters are adjusted in a way to only favor strong signals like the reflective materials on the drogue and signals returned from the carpet floor in the conference room during ground test are too weak to pass the threshold test in Pseudo Codes Line 7. Fortunately, those distractions are not expected in the flight scenarios. One may notice in T3, T4 and T5 data sets, there are perceptible defect pixels in the sensor where no range or intensity values are reported. We intentionally employed this non-perfect camera to perform this ground test to better evaluate the robustness of the center estimation module under more practical conditions. Also, in real world scenarios, a full circle may not be detected due to the drogue partially out of field of view, or occlusion by the prober, or imaging a used drogue that has spots covered with spilled fuel, resulting in lower reflected signal returns than normally expected. We are pleased to find the circle fitting based algorithm is, in fact, more forgiving, and potentially can be integrated in a deployed system.

As can be seen in the center subgraph of Figure 17, all 3D points captured at different times lie within the 30° FOV boundary because the control logic of the robot was designed to align the estimated drogue center at the center of the image while continuously shortening the estimated range to the target. Although during the test, partially outside field of view cases like Figure 14c may occur occasionally (or intentionally during evaluation) the ground robot makes a proper course correction in the next frame and brings the target back to the area close to the image center. The ground navigation robot always catches the drogue during the final docking step (or hitting the basket step) for all test runs using the miss and catch criteria defined in AARD project. The ground test demonstrated in this paper, however, is a simplified evaluation, and it is expected to have a much more sophisticated navigation and control development effort to carry out a similar test in the air—Not to mention additional turbulence conditions and aircraft generated aerodynamics complications which were all omitted from the ground test. A successful ground demonstration using an autonomous system is an encouraging step toward the logical subsequent flight evaluation. Although the algorithm implemented on the ground robot does not use any information from the previous image frame (target detection only, non-tracking), the proposed method is not limited to isolated frames. Instead, use of past information is highly recommended for trajectory prediction and course smoothing purposes, especially in the flight test.

The goal of the proposed sensor-in-the-loop approach is to consider data acquisition performed by sensor hardware and image processing carried out by software algorithms simultaneously. To optimize the system as a whole for a specific application, partitioning tasks between the hardware and software components, using their complementary strengths, is essential. This paper suggests one combination: lowest gain and highest bandwidth setting in the sensor with a circle fitting algorithm, to estimate the 3D position of the center of the drogue for the autonomous aerial refueling application. It is possible and advantageous to make a more intelligent system by adaptively varying parameters on-the-fly, such as with automatic gain control (AGC) and selecting appropriate data processing algorithms depending on the observed scenery. These interesting, yet challenging, topics deserve further research.

## 4. Conclusions

A sensor-in-the-loop, non-tracking approach is proposed to address the probe and drogue (PDR) style autonomous aerial refueling task. By successfully using a surrogate robot to perform the final docking stage of the aerial refueling task on the ground, the experimental results suggest that applying computer vision fault tolerant circle fitting algorithms on images acquired by a 3D Flash LIDAR camera with lowest gain and highest bandwidth settings has great potential to reliably measure the orientation and relative position between the drogue and the prober for unmanned aerial refueling applications. To the best of our knowledge, we are the first group to demonstrate the feasibility of using a camera-like time-of-flight based sensor on an autonomous system. The sensor-in-the-loop design concept seeks an optimum solution by balancing tasks between the hardware and software components, using their complementary strengths, and is well-suited to solve challenging problems for future autonomous systems. This paper concludes a successful ground test and we are looking for opportunities to further verify the proposed method in-flight and eventually deploy the solution.
